# Aflatoxin in Chili Peppers in Nigeria: Extent of Contamination and Control Using Atoxigenic *Aspergillus flavus* Genotypes as Biocontrol Agents

**DOI:** 10.3390/toxins11070429

**Published:** 2019-07-22

**Authors:** Chibundu N. Ezekiel, Alejandro Ortega-Beltran, Eniola O. Oyedeji, Joseph Atehnkeng, Philip Kössler, Folasade Tairu, Irmgard Hoeschle-Zeledon, Petr Karlovsky, Peter J. Cotty, Ranajit Bandyopadhyay

**Affiliations:** 1 International Institute of Tropical Agriculture, Ibadan 200001, Nigeria; 2 Department of Microbiology, Babcock University, Ilishan Remo 121103, Nigeria; 3 National Horticultural Research Institute (NIHORT), Ibadan 200272, Nigeria; 4 Molecular Phytopathology and Mycotoxin Research Section, University of Gottingen, 37073 Gottingen, Germany; 5 USDA-ARS, Tucson, AZ 85701, USA; 6 Present address of Peter Cotty: P.O. Box 65699, Tucson, AZ 85728, USA

**Keywords:** aflatoxin biocontrol, atoxigenic strains, chili peppers, fungal community structure

## Abstract

Across sub-Saharan Africa, chili peppers are fundamental ingredients of many traditional dishes. However, chili peppers may contain unsafe aflatoxin concentrations produced by *Aspergillus* section *Flavi* fungi. Aflatoxin levels were determined in chili peppers from three states in Nigeria. A total of 70 samples were collected from farmers’ stores and local markets. Over 25% of the samples contained unsafe aflatoxin concentrations. The chili peppers were associated with both aflatoxin producers and atoxigenic *Aspergillus flavus* genotypes. Efficacy of an atoxigenic biocontrol product, Aflasafe, registered in Nigeria for use on maize and groundnut, was tested for chili peppers grown in three states. Chili peppers treated with Aflasafe accumulated significantly less aflatoxins than nontreated chili peppers. The results suggest that Aflasafe is a valuable tool for the production of safe chili peppers. Use of Aflasafe in chili peppers could reduce human exposure to aflatoxins and increase chances to commercialize chili peppers in premium local and international markets. This is the first report of the efficacy of any atoxigenic biocontrol product for controlling aflatoxin in a spice crop.

## 1. Introduction

In most sub-Saharan Africa (SSA), chili peppers are an important ingredient to prepare a large array of traditional dishes [1]. Consumption of high quantities of chili peppers is associated with health benefits, including disease prevention [2,3]. However, chili peppers in SSA are frequently contaminated with aflatoxins by *Aspergillus* section *Flavi* fungi [4,5]. Aflatoxins are highly toxic and carcinogenic mycotoxins. Even at minute concentrations, aflatoxins pose serious health threats to humans. Immunosuppression, growth retardation, liver diseases, and cancer are among the health effects that may occur after consumption of crops contaminated with unsafe aflatoxin levels [6,7,8]. Consumption of high aflatoxin concentrations can lead to liver necrosis followed by rapid death [9,10]. Chili pepper consumption may contribute to chronic human aflatoxin exposure.

Other aflatoxin-prone crops include maize, groundnut, sorghum, and tree nuts. Crop infection by aflatoxin producers and subsequent aflatoxin contamination may occur in the field and continue during storage [11]. In addition to severe health problems, aflatoxins impede trade and economic growth [12,13]. Crops exceeding tolerance thresholds are banned in nations where aflatoxins are strictly regulated. This results in lost export opportunities [12,14]. Every year, shipments are rejected at European borders because of high aflatoxin concentrations [15]. A recent example is the rejection of chili peppers from Ethiopia worth over 10 million USD [16]. The adverse health effects and economic barriers caused by these dangerous toxins demand tailored, well-designed aflatoxin management strategies [17]. Those strategies must involve technologies preventing aflatoxin contamination at both pre- and post-harvest stages [18,19]. An initial step in designing proper aflatoxin management strategies is assessment of the distribution and severity of the problem in a target area/nation.

The most frequently identified causal agent of aflatoxin contamination across the globe is *A. flavus* [20,21]. There are two main morphotypes within *A. flavus*. The L morphotype produces copious conidiation, large sclerotia (dia > 400 μm), and variable levels of B aflatoxins (some producing no aflatoxins) while the S morphotype produces scarce conidiation, numerous small sclerotia (dia < 400 μm) and consistently high concentrations of B aflatoxins [22]. In West Africa, *Aspergillus* section *Flavi* members with S morphology are common in soils [23] and associated with maize, groundnut, sesame, and chili, and are referred to as S_BG_ because they produce both B and G aflatoxins [24,25,26,27,28]. Molecular phylogenetics recently has been used to divide these S_BG_ members of section *Flavi* resident in West Africa into several taxa including *A. aflatoxiformans*, *A. austwickii*, *A. cerealis*, *A. minisclerotigenes*, and unnamed taxa [27,29,30]. *A. aflatoxiformans* and *A. minisclerotigenes* appear to be more common across the sub-region. In the current study, the term S_BG_ strains is used for all S-morphotype fungi producing both B and G aflatoxins. All S_BG_ taxa resident in Nigeria produce high concentrations of aflatoxins (>300 parts per billion [ppb]) [23,27] and should be considered important causal agents of contamination, even when found at relatively low frequencies [25,31].

Some genotypes within the *A. flavus* L morphotype contain genetic defects in the aflatoxin biosynthesis gene cluster that prevent them from producing aflatoxins. Those genotypes are known as atoxigenic. When carefully selected and tested, atoxigenic genotypes can be used as biocontrol agents to limit crop aflatoxin content [32]. Aflatoxin biocontrol using atoxigenic fungi is a technology developed by the United States Department of Agriculture—Agricultural Research Service (USDA–ARS) [33]. The technology has been used for decades in the US where it is registered for aflatoxin mitigation of cottonseed, maize, pistachio, almond, fig, and groundnut [33,34,35,36]. The International Institute of Tropical Agriculture (IITA), USDA–ARS, and several partners have adapted and improved the technology for use in Nigeria and other SSA nations under the trade name Aflasafe [17,37]. In Nigeria, the biocontrol product Aflasafe^TM^ is registered with the National Agency for Food and Drug Administration and Control (NAFDAC) for use in maize and groundnut.

Chili peppers are important components of Nigerian cuisine with large quantities used in traditional dishes including suya, tsire, kilishi, and pepper soup. Globally, Nigeria is the 5th and 11th largest producer of green and dry chili peppers, respectively [38]. Despite significant production, consumption, and export potential of chili peppers in Nigeria, there is limited information on aflatoxin contamination of Nigeria’s chili peppers [39]. Relatively recently, a study comparing aflatoxin concentrations in chili peppers produced and marketed in Nigeria with chili peppers originating from various countries and sold in US markets was published [4]. The examination of 55 chili pepper samples from Nigeria collected in local, small-scale markets in Lagos and Kaduna states, revealed that chili pepper consumption is a potentially significant route of local exposure to aflatoxins. Due to the importance of chili peppers, additional studies are needed to determine distribution of chili pepper aflatoxin contamination in Nigeria, including the major chili pepper producing areas. It is also necessary to characterize the fungal communities associated with the chili peppers as well as their aflatoxin-producing potentials. In addition, chili pepper farmers would benefit if the biocontrol product Aflasafe is found to be effective to limit aflatoxin contamination in the crop. Thus, the current study sought to: (i) quantify aflatoxin concentrations in chili peppers grown in three agroecological zones (AEZ) of Nigeria, (ii) characterize the aflatoxin-producing fungi associated with the chili peppers, and (iii) test the field efficacy of Aflasafe in limiting aflatoxin contamination of chili peppers. This is the first report across the globe of the use of an aflatoxin biocontrol product for limiting chili pepper aflatoxin content. Results from the current study will aid to design aflatoxin management strategies tailored for chili pepper, a crop widely grown and consumed in Nigeria. Treatment of chili peppers with Aflasafe has the potential to allow production of aflatoxin-compliant chili peppers, significantly reduce human aflatoxin exposure among frequent consumers of the spice, and unlock premium markets for chili pepper farmers.

## 2. Results

### 2.1. Aflatoxin Contamination of Chili Pepper from Farmers’ Stores and Markets

A total of 70 chili pepper samples were collected from farmers’ stores and local markets in three states of Nigeria (Table 1). The majority (69%) of the chili pepper samples were contaminated with aflatoxins (mean = 8.9 ± 1.9 ppb; Table 2). No aflatoxins were detected in 22 of the examined samples. Aflatoxin levels in chili peppers from Oyo (mean = 15.2 ppb) were significantly (*p* < 0.05) higher than those from Kano (mean = 6.0 ppb) and Nasarawa (mean = 5.1 ppb). In Oyo, 29.2% of the samples had more than 5 ppb aflatoxin B_1_ and 45.8% had more than 10 ppb total aflatoxin, the EU maximum limit. When samples were examined by source, one-third of chili peppers from urban markets had total aflatoxin levels above 10 ppb. However, there were no significant differences in aflatoxin content among the examined sources.

### 2.2. Incidence and Densities of Aspergillus Section Flavi

A total of 1380 isolates of *Aspergillus* section *Flavi* were recovered from all the chili pepper samples (Table 3). The *A. flavus* L morphotype dominated the communities in each state (range = 85.7%–88.7%) and each source (range = 78.6%–95.3%). Frequencies of the L morphotype and *A. tamarii* did not differ (*p* > 0.05) independently within state or source. Other recovered fungi included S_BG_ strains. Significantly (*p* < 0.05) higher frequencies of S_BG_ strains were detected in chili peppers from farmers’ stores than in chili peppers from urban markets (Table 3). The frequency of S_BG_ strains was similar across states. The frequencies of both S_BG_ strains and *A. tamarii* were always lower (*p* < 0.05) than those of the L morphotype (Table 3). Significant differences were detected in fungal densities (colony-forming units (CFU)/g) by state and source (Table 3). Chili peppers from Oyo had higher (*p* < 0.05) CFU/g than those collected from Kano, while samples from farmers’ store had significantly less (*p* < 0.05) CFU/g than samples from urban market.

### 2.3. Aflatoxin-Producing Ability of the Recovered Fungi

**Figure 1:**
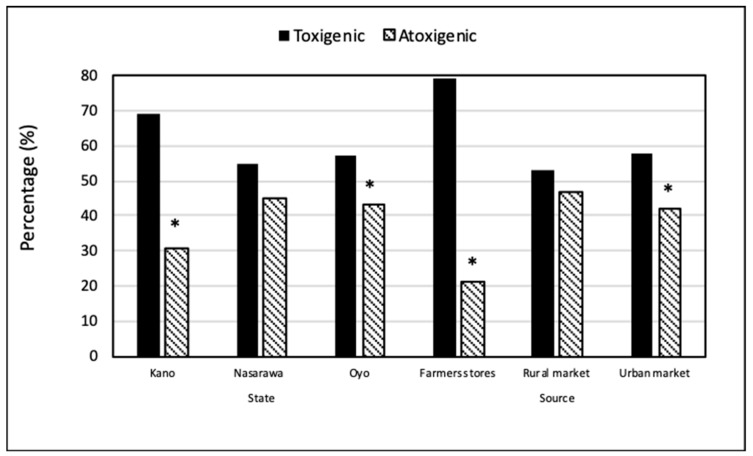
Proportions of aflatoxin-producers and atoxigenic isolates in *Aspergillus* section *Flavi* associated with chili peppers collected in three states of Nigeria, from three sources. Bars with an asterisk (*) denote significant differences in the examined state or source (Student’s *t*-test, α = 0.05).

Out of the 1380 *Aspergillus* isolates, 828 isolates produced varying aflatoxin levels on colonized maize grains in laboratory assays (Figure 1). All 111 S_BG_ strain isolates and 717 of the 1207 *A. flavus* L morphotype isolates produced aflatoxins on the maize grains. As expected, none of the *A. tamarii* isolates produced aflatoxins. Frequencies of aflatoxin producers were higher than those of atoxigenic isolates (Figure 1). Significant (*p* < 0.05) differences were detected between proportions of aflatoxin-producers and atoxigenic isolates in Kano and Oyo, and in farmers’ stores and urban markets.

### 2.4. Aflatoxin Biocontrol in Chili Peppers Using the Product Aflasafe

Proportions of each type of fungus and overall fungal densities were examined in treated and control plots before Aflasafe application, at harvest, and six months after Aflasafe application. In general, proportions of each type of fungus and fungal densities were statistically similar for all treatments (Table 4) except for frequencies of both the L morphotype and S_BG_ strains six months after Aflasafe application. Generally, the frequencies of L morphotype were higher (*p* < 0.05) in treated fields while frequencies of S_BG_ strains were higher (*p* < 0.05) in control fields. The *A. flavus* L morphotype dominated fungal communities in the three states in both treated and control fields, in all three examined substrates (Table 4). In Kano, S_BG_ strains occurred at low levels (<5%) in soils before treatment but composed over 13% of the communities in treated and nontreated chili pepper at harvest. In Oyo and Nasarawa, the frequencies of S_BG_ strains were low (<5%) at harvest (Table 4). Six months after application, in all states, S_BG_ strains were important members of the *Aspergillus* communities (range = 14.2%–40.6%). Frequencies of *A. tamarii* were generally low. Although nonsignificant, there was a 51-fold increase in fungal densities in treated chili pepper in Kano (Table 4). On the other hand, fungal densities in control chili peppers were 19 times higher than those of treated chili peppers in Oyo (Table 4).

**Figure 2:**
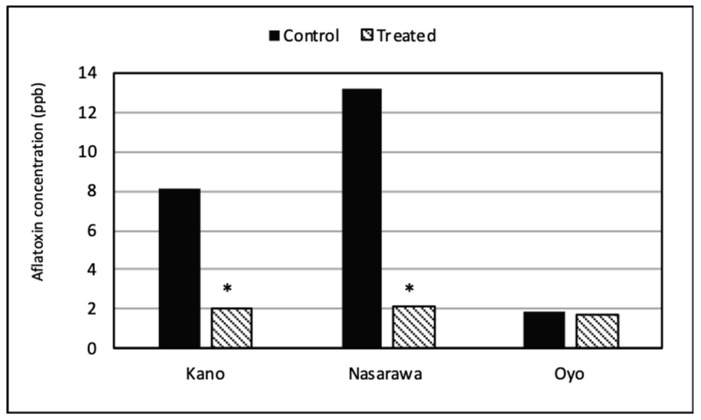
Mean aflatoxin concentrations in chili pepper at harvest from Aflasafe-treated and control fields in three states of Nigeria. Bars with an asterisk (*) denote significant differences between treated and control chili peppers (Student’s *t*-test, α = 0.05).

Overall, the aflatoxin content in treated chili pepper was lower than in nontreated (control) chili peppers (Figure 2). Low aflatoxin levels were detected in both treated (mean = 1.7 ppb) and untreated chili pepper (mean = 1.9 ppb) from Oyo. High and significant (*p* < 0.05) aflatoxin reductions compared to controls were observed in Aflasafe-treated chili peppers from Kano and Nasarawa (75.6% and 84.2% less aflatoxins, respectively).

Relatively low incidences of the four atoxigenic African *Aspergillus flavus* vegetative compatibility groups (AAVs) composing Aflasafe were recorded from both treated and control soils before Aflasafe application (range = 3.1%–10.4%; Table 5). Incidence of the Aflasafe AAVs in treated chili peppers ranged from 8.9% to 25.0% while in nontreated chili peppers the range was 6.9% to 9.4%. Higher (*p* < 0.05) incidence of Aflasafe AAVs were detected in treated chili peppers from Kano and Nasarawa. When soils were examined six months after application, high incidence of the four Aflasafe AAVs was detected in treated soils (range = 55.4%–67.0%) compared to nontreated soils (range = 4.5%–7.2%).

## 3. Discussion

Despite the great importance of chili pepper in traditional cuisine across SSA, there are relatively few reports of aflatoxin levels found in the crop [4]. In addition, the information regarding appropriate aflatoxin management strategies to limit chili pepper contamination in Nigeria, and throughout West Africa, is very limited. In the current study, aflatoxin levels in chili pepper from three states of Nigeria were documented. The results agree with recent observations on fresh chili peppers from the states of Kaduna and Lagos that aflatoxin contamination of peppers is widely distributed in Nigeria with over 65% of peppers contaminated and 25% of the contaminated peppers containing concentrations above the 10 ppb total aflatoxin tolerance threshold established by the EU [4,38]. These results suggest that consumption of dried chili pepper is a potentially important avenue for chronic aflatoxin exposure in human populations of Nigeria given the importance of chili peppers in Nigerian cuisine. With respect to variations in aflatoxin concentrations among the examined samples, we detected higher aflatoxin levels in chili peppers from Oyo than from Kano and Nasarawa. Most of the chili pepper sold in Oyo markets are produced in northern half of Nigeria and then transported to Oyo and other states in the south of the country. Postharvest practices influence crop aflatoxin contamination [11] and factors contributing to higher aflatoxin content in chili peppers sold in Oyo may include length of transportation, poor transportation conditions, and inadequate storage conditions prior to end-use. Oyo falls in a wet, humid, and warm ecological zone where conditions are favorable for postharvest aflatoxin formation. 

The current study also examined the fungal communities associated with the chili peppers. The majority of recovered *Aspergillus* section *Flavi* (60%) produced aflatoxins in maize inoculation studies (Figure 1). People in other SSA nations may also be exposed to unsafe aflatoxin concentrations through consumption of chili peppers given that the implicated aflatoxigenic fungal species are common residents in many of the countries [24,25,26,28] and that Nigerian chili peppers are exported to neighboring countries. Moreover, sale of chili pepper from Nigeria into premium markets is hindered by frequencies of aflatoxin contamination detected in the current study. The incidence of S_BG_ strains was higher in Kano compared to Nasarawa and Oyo. Kano is in the far north center of Nigeria where the climate is hotter and dryer than in the two other states. Previous reports examining fungal communities associated with maize and maize field soil have documented relatively high incidences of S_BG_ strains in dryer, hotter regions of Nigeria [23,25,40]. Surprisingly, S_BG_ strains were found at low levels (<5%) in soils before treatment in the state of Kano, a state where this type of fungi is known to thrive. 

Not all *Aspergillus* species associated with a given crop produce aflatoxins. Among the most common aflatoxin producer, *A. flavus*, there are genotypes lacking the ability to produce aflatoxins. Those isolates are known as atoxigenic [11]. We detected a relatively large number of atoxigenic *A. flavus* genotypes in the examined samples. Atoxigenic strains can be used in biocontrol formulations to displace aflatoxin producers when applied in the field and reduce crop aflatoxin content [11,17,32]. The biocontrol product Aflasafe was applied in chili pepper fields at Kano, Nasarawa, and Oyo states during a single cropping season. The natural occurrence of the four atoxigenic AAVs composing Aflasafe in chili pepper production in the three examined states (Table 5) indicates that the four AAVs are relatively common and adapted to these chili pepper agroecosystems. Use of native, well-adapted atoxigenic strains is one of the principles of the atoxigenic biocontrol technology [32]. The four Aflasafe genotypes were originally detected in maize agroecosystems of Nigeria [40,41]. Loss of premium markets for chili pepper due to aflatoxin contamination may be mitigated through use of the biocontrol product Aflasafe which was shown in the current study to effectively reduce aflatoxins in chili peppers (Figure 2). The lower, safer aflatoxin concentrations in chili pepper from fields treated with Aflasafe compared to nontreated controls in Kano and Nasarawa provides hope for peppers with improved value and greater health benefit in Nigeria and other production areas.

Efficacy of biocontrol has been previously reported in cottonseed, maize, and a few nut crops [17,33,34,35,36]. This is the first report of field efficacy of an atoxigenic aflatoxin biocontrol product for management of aflatoxins in any spice crop. In the US, the aflatoxin biocontrol product AF36 Prevail is effective in multiple crops [33,34,35]. In Nigeria too, a single biocontrol product is effective in controlling aflatoxins in maize, groundnut [17], and chili pepper, as we report in this paper. Our findings will help in obtaining approval from the Nigerian regulatory authorities for extending the commercial use of the registered product Aflasafe to chili pepper in addition to maize and groundnut in Nigeria.

Efficacy of aflatoxin biocontrol is most readily assessed in regions and during seasons when the risk of aflatoxin contamination is high. Lower aflatoxin concentrations were detected in chili peppers treated with Aflasafe from Kano and Nasarawa compared to nontreated fields where aflatoxin concentrations exceeded 8 ppb. In Oyo, aflatoxin concentrations in controls were low (<2 ppb) and differences between treated and control fields could not be resolved. Low incidences of atoxigenic AAVs occurred in chili peppers from treated fields at harvest compared to results from Aflasafe treatment of maize and groundnut [17,37,41]. This may have been due to the varying flowering stages of chili peppers at the time of treatment. Results from the current study are encouraging and suggest that atoxigenic biocontrol agents can reduce incidences and severities of aflatoxin contamination in chili pepper in a manner similar to that achieved for maize, groundnut, cottonseed, pistachio, fig, and almond [17,33,34,41,42,43]. However, studies directed at optimizing timing and method of applications to chili pepper are needed. Exploration of benefits of rotation with other treated crops and the synergisms achieved by carry-over of the biocontrol fungi between crops [32,33,41,44] should also be pursued.

Treating chili peppers with a biocontrol product may aid in reducing human exposure to aflatoxins and increasing the market value of treated crops. Aflasafe is approved by NAFDAC, the Nigerian biopesticide regulator, for use in maize and groundnut throughout the country. Registration of the technology for use in chili peppers is pending in Nigeria. Upon successful completion of the registration process, producers of chili peppers could benefit if they use the biocontrol technology together with good drying and storage practices. Biocontrol as an aflatoxin management option should be explored in other countries where atoxigenic biocontrol products are available and aflatoxin contamination of chili peppers is a perennial problem.

## 4. Conclusions

Aflatoxin contamination of staple food continues to challenge global food security, especially in high-risk regions such as SSA. In the current study, aflatoxin contamination of chili peppers is shown to be frequent in Nigeria, and, as such, a potentially important contributor to the cumulative chronic aflatoxin exposure that residents in that nation experience. More importantly, we have presented snapshot data indicating that the aflatoxin biocontrol product Aflasafe, which is gaining widespread attention and adoption across SSA, is a useful management tool for reducing aflatoxin content in chili peppers in the field and consequent human exposure. In view of this first report on the field efficacy of Aflasafe towards aflatoxin mitigation in chili pepper, we propose the following next steps: (a) multi-year, multi-site evaluation of the field efficacy of Aflasafe on aflatoxin reduction in chili peppers, (b) evaluation of the impact of storage conditions (facility and duration) on aflatoxin accumulation in chili peppers, (c) assessment of the effect of Aflasafe on aflatoxin reduction during chili pepper storage, and (d) development of market linkages between producers of Aflasafe-treated chili peppers and industries searching for aflatoxin-compliant chili peppers. Furthermore, future research ideas to consider on the broader scale include evaluating the impact of Aflasafe on the reduction of aflatoxin exposure in human populations using biological markers of exposure.

## 5. Materials and Methods

### 5.1. Collection of Chili Samples for Baseline Study

A total of 70 dried chili pepper samples (~2 kg each) from farmers’ stores/households, urban markets, and rural markets were collected across three states in Nigeria: Kano, Nasarawa, and Oyo. Those states were selected to reflect high, medium, and low chili pepper producing states, respectively. The number of samples and the source per state is given in Table 1. Samples were transported to IITA’s Pathology Laboratory and immediately dried in a forced-air oven at 50 °C for 2 d. After drying, samples were individually ground using a laboratory blender (Waring Commercial, Springfield, MO, USA) for 30 s in a 110 mL stainless steel blending jar (MC-2) and thoroughly mixed. Ground homogenized samples were divided into two equal portions (~1 kg each), one for microbial analyses was stored at 4 °C and the other for aflatoxin analyses was stored at −20 °C before analyses. Samples were placed in sealed plastic bags prior to cold storage. The blending jar was washed between samples with 80% ethanol to prevent cross-contamination by both microorganisms and aflatoxins.

### 5.2. Aspergillus Associated with the Chili Peppers

Community structures of aflatoxin-producing fungi associated with chili peppers were determined by placing all the recovered *Aspergillus* section *Flavi* fungi into their corresponding species and/or morphotypes. Fungi were recovered by dilution plate technique on modified Rose Bengal Agar (MRBA) [45]. Briefly, 1 g of each sample was suspended in 10 mL sterile distilled water contained in a 40 mL sterile polystyrene tube, vortexed for 30 s, and plated on MRBA in triplicate. Plates were incubated for 3 d (31 °C, dark). Plates with over 10 *Aspergillus* section *Flavi* colonies were discarded and the process was repeated using appropriate dilutions. For each sample, 10 putative section *Flavi* isolates were transferred to 5-2 agar (5% V-8 juice (Campbell Soup Company, Camden, NJ, USA)), 2% Bacto agar ((Difco Laboratories Inc., Detroit, MI, USA), pH 6.0) and incubated for 5 d (31 °C). Members of *Aspergillus* section *Flavi* were identified based on colony characteristics, spore ornamentation, and aflatoxin production profile (see below for aflatoxin quantification) [25,29,46]. Densities of *Aspergillus* section *Flavi* fungi were calculated as colony-forming units (CFU) per g of sample. All recovered isolates were maintained as agar plugs (3-mm dia) of sporulating cultures in 2 mL sterile distilled water as working cultures. In addition, all isolates were stored on silica gel at 4 °C for long term storage [47].

### 5.3. Aflatoxin Analysis of Chili Samples

The batch of chili pepper samples intended for aflatoxin analyses was sent to the University of Gottingen, Germany, for aflatoxin quantification using high-performance liquid chromatography (HPLC). All samples were shipped as dry powder. Aflatoxins were extracted from the samples with acetonitrile/water (84:16; solvent/sample ratio of 10:1 (*v*/*w*)). The extracts were cleaned by solid-phase extraction on Puritox columns (R-Biopharm GmbH, Darmstadt, Germany) and solvent was removed in vacuo. The residue was dissolved and derivatized in 100 µL of trifluoroacetic acid for 15 min at RT, diluted 10-times with acetonitrile/water (10:90), and filtered and injected into a reverse-phase HPLC system described previously [48]. Aflatoxins were detected by fluorescence using excitation at 365 nm and emission at 450 nm [49,50,51] and quantified using matrix-matched standards prepared using commercial analytical standards (Sigma-Aldrich GmbH, Steinheim, Germany).

### 5.4. Aflatoxin-Production Ability of the Recovered Fungi

Aflatoxin-producing potentials of all 1380 *Aspergillus* section *Flavi* isolates recovered in the baseline study were evaluated in autoclaved maize kernels, as described by Atehnkeng et al. [44]. Briefly, 5 g of healthy, aflatoxin-free maize kernels were placed into 40 mL polystyrene vials and washed with two changes of tap water. This was followed by soaking of the kernels in 25 mL tap water overnight to allow imbibing adequate moisture for fungal growth. After decanting excess water, kernels were washed once in tap water and then autoclaved at 121 °C for 20 min. For each isolate, cooled kernels were independently inoculated with 500 μL of a suspension containing approximately 10^6^ spores and incubated for 7 d at 31 °C. Kernels inoculated with 500 µL of sterile distilled water served as negative controls. Aflatoxins were extracted and quantified using thin layer chromatography and scanning densitometry as described by Atehnkeng et al. [44]. Isolates that did not produce detectable aflatoxins (limit of detection: 0.5 ppb) were classified as atoxigenic.

### 5.5. Preparation and Quality Control of Aflasafe

Aflasafe was produced in IITA-Ibadan, Nigeria, using a laboratory-scale method previously described [41]. To prepare Aflasafe, batches of autoclaved sorghum grain were individually inoculated with a spore suspension of the four atoxigenic AAVs type isolates. This was repeated separately for each of the four atoxigenic AAVs that compose the biocontrol product. The type isolates were La3279, La3304, Ka16127, and Og0222. Inoculated sorghum was incubated at 31 °C for 18 h, and dried in an oven at 55 °C for 4 d. Equal proportions of dried grains separately inoculated with each of the four atoxigenic isolates were mixed. The finished formulated product was placed in 2.5 kg polyethylene bags and stored at ambient temperature until used.

The quality (purity, sporulating ability, and composition of the active ingredient fungi) of the finished product was determined in the laboratory. For each 20 kg, approximately 100 g of product was collected and transferred to sterile plastic bags. In a biological safety cabinet, 100 sorghum grains of each sample were plated onto two plates each of 5-2 agar, Nutrient Agar (Lab M, UK; 28 g/L, 20 g/L glucose), and Violet Red Bile Agar (VRBA; Difco Laboratories, 41.5 g/L, pH 7.4). Plates were incubated at 31 °C for 7 d. After incubation, the number of grains colonized by *A. flavus* and presence/absence of any other microorganism, including fecal coliforms on VRBA, were recorded. Spore production and isolate membership in one of the four Aflasafe AAVs were evaluated using protocols previously described [40,41].

### 5.6. Field Plots and Aflasafe Application

The efficacy of Aflasafe in limiting aflatoxin contamination in chili peppers was evaluated in farmers’ fields at Kano, Nasarawa, and Oyo states during the cropping season of 2012. Aflasafe was deployed in collaboration with members of farmers’ associations. Most farmers voluntarily consented to conduct the efficacy trials but some farmers in Oyo requested monetary incentives to participate in the trials. Farmers grew the chili peppers according to their own agronomic practices. Farmers weeded the fields by hand or bullocks, top-dressed with urea, and earthed-up prior to application of Aflasafe to avoid burying the product. Aflasafe was broadcasted by hand once at a rate of 10 kg/ha 2–3 weeks before initiation of crop flowering. Farmers received training on how to apply Aflasafe as described by Atehnkeng et al. [41]. For each Aflasafe-treated field, two neighboring fields more than 500 m apart were selected as corresponding untreated control fields. The number of Aflasafe-treated and control fields is given in Table 4. Field size was on an average 0.6 ha. All fields were rainfall dependent.

### 5.7. Soil and Crop Sampling from Field Plots

Approximately 150 g of soil was collected from each control and treated field before and six months after Aflasafe application. Each soil sample consisted of ~50 subsamples taken to a depth of 2 cm from three random locations [52]. Samples were placed in labeled paper bags and, after arrival in the laboratory, were dried in a forced-air oven (48 h, 50 °C). After drying, samples were transferred to a biological safety cabinet and soil clods were broken with a hammer. Samples were homogenized by hand within polyethylene plastic bags.

At harvest, around 2 kg of fresh chili pepper fruits were collected from all treated and untreated fields. Chili peppers were transported to the laboratory, sun-dried for 3–4 d, and processed and stored as described in Section 5.1 until used in chemical and microbial analyses.

### 5.8. Aspergillus Species Associated with Aflasafe-Treated and Untreated Chili Peppers

*Aspergillus* section *Flavi* were isolated and assigned to the corresponding fungal type as described in Section 5.2. Frequencies of the four constituent AAVs of Aflasafe were determined using protocols previously described [40,41].

### 5.9. Aflatoxin Analyses of Aflasafe-Treated and Control Chili Pepper

Half a portion (~1 kg) of all treated and control chili pepper samples (*n* = 78) were sent to the University of Gottingen, Germany, for aflatoxin analyses. Aflatoxin quantification was conducted as described in Section 5.3.

### 5.10. Statistical Analyses

Data analyses were performed using SPSS v.15 (SPSS^®^ Inc., Chicago, IL, USA). Means were calculated and tested for significance by the one-way analysis of variance test at α = 0.05 or by the Students *t*-test at α = 0.05 as appropriate. Mean separations were performed with Duncan’s Multiple Range Test (DMRT).

## Figures and Tables

**Table 1:** Origin of chili pepper samples examined in the current study.

Sample Source	States		
	Kano	Nasarawa	Oyo
Farmer store	10	4	0
Rural markets	8	14	13
Urban markets	5	5	11
Total	23	23	24

**Table 2:** Aflatoxin levels in chili pepper samples collected in three states of Nigeria, from three sources.

Origin	N ^a^	Aflatoxin Levels (ppb) with Respect to EU Limits				
		% ^b^	Total Aflatoxin Range	Mean ± SE ^c^	Aflatoxin B_1_ >5 ppb (%)	Total Aflatoxin >10 ppb (%)
State						
Kano	23	70	0–16	5.1 ± 1.0 b	0 (0.0)	3 (13.0)
Nasarawa	23	48	0–59	6.0 ± 2.8 b	2 (8.7)	3 (13.0)
Oyo	24	88	0–97	15.2 ± 4.6 a	7 (29.2)	11 (45.8)
Source						
Farmers’ store	14	71	0–18	6.4 ± 1.7 a	0 (0.0)	3 (21.4)
Rural market	35	66	0–72	8.3 ± 2.6 a	6 (17.1)	7 (20.0)
Urban market	21	71	0–97	11.3 ± 4.6 a	3 (14.3)	7 (33.3)

^a^ Number of chili pepper samples. ^b^ % samples contaminated with aflatoxins. ^c^ Means followed by same letter are not significantly different by DMRT (α = 0.05); states and sources were compared separately.

**Table 3:** Community compositions of aflatoxin-producing fungi associated with chili peppers collected in three states of Nigeria, from three sources.

Origin and Source	N ^a^	Recovered Isolates	Proportion of Species (%) ^b^			CFU/g
			*A. flavus* L morphotype	S_BG_ Strains	*A. tamarii*	
State						
Kano	23	440	85.7 ± 1.3 aA	13.6 ± 1.3 aB	0.7 ± 0.1 aB	12,183 b
Nasarawa	23	460	88.7 ± 1.1 aA	9.1 ± 1.1 aB	2.2 ± 0.3 aB	27,100 ab
Oyo	24	480	87.9 ± 1.1 aA	1.9 ± 0.3 aB	10.2 ± 1.1 aB	73,012 a
Source						
Farmers’ store	14	280	78.6 ± 2.0 aA	20.0 ± 2.0 aB	1.4 ± 0.1 aB	10,293 b
Rural market	35	700	86.6 ± 1.0 aA	6.4 ± 0.7 abB	7.0 ± 0.7 aB	31,446 ab
Urban market	21	400	95.3 ± 0.4 aA	2.5 ± 0.4 bB	2.3 ± 0.2 aB	67,195 a

^a^ Number of chili pepper samples. ^b^ Means followed by the same letter are not significantly different by DMRT (α = 0.05); states and sources were compared separately. Lower-case letters compare data (proportion of each species or CFU/g) among states or sources (i.e., within columns). Upper-case letters compare frequencies of the aflatoxin-producing fungal types by state and source (i.e., across rows).

**Table 4:** Frequencies of *Aspergillus* fungi in soil before Aflasafe application, chili pepper at harvest, and soil collected six months after Aflasafe application in biocontrol field efficacy trials in three states of Nigeria.

Substrate	Treatment	N ^a^	Proportion of Species (%) ^b^			CFU/g ^b^
			*A. flavus* L morphotype	S_BG_ Strains	*A. tamarii*	
Kano						
Soil before application	Aflasafe	10	96.6 ± 0.0 ^ns^	1.7 ± 0.1 ^ns^	1.7 ± 0.1 ^ns^	99 ^ns^
	Control	20	99.1 ± 0.5	0.6 ± 0.4	0.3 ± 0.3	143
Chili pepper at harvest	Aflasafe	10	80.0 ± 9.8 ^ns^	13.8 ± 8.8 ^ns^	6.1 ± 3.8 ^ns^	4,940,181 ^ns^
	Control	20	83.8 ± 6.0	16.3 ± 6.0	0.0	97,755
Soil 6 mo. after application	Aflasafe	10	77.9 ± 0.3 ^ns^	20.5 ± 1.5 ^ns^	1.6 ± 0.3 ^ns^	405 ^ns^
	Control	20	66.3 ± 0.9	30.0 ± 1.2	3.7 ± 0.5	382
Nasarawa						
Soil before application	Aflasafe	7	92.2 ± 0.0 ^ns^	1.7 ± 0.2 ^ns^	6.2 ± 0.9 ^ns^	167 ^ns^
	Control	14	93.8 ± 0.0	3.9 ± 0.4	2.3 ± 0.2	80
Chili pepper at harvest	Aflasafe	7	99.2 ± 0.8 ^ns^	0.0 ^ns^	0.8 ± 0.8 ^ns^	626,086 ^ns^
	Control	14	99.6 ± 0.4	0.0	0.4 ± 0.4	923,825
Soil 6 mo. after application	Aflasafe	7	76.1 ± 0.0 *	21.0 ± 1.7 *	2.9 ± 0.3 ^ns^	379 ^ns^
	Control	14	55.2 ± 1.2	40.6 ± 1.8	4.2 ± 0.4	659
Oyo						
Soil before application	Aflasafe	9	98.8 ± 0.0 ^ns^	0.0 ^ns^	3.7 ± 0.2 ^ns^	377 ^ns^
	Control	18	94.5 ± 0.0	4.5 ± 0.7	0.9 ± 0.1	159
Chili pepper at harvest	Aflasafe	9	96.1 ± 2.8 ^ns^	2.7 ± 2.1 ^ns^	1.0 ± 0.8 ^ns^	45,256 ^ns^
	Control	18	97.4 ± 1.4	2.0 ± 1.2	0.6 ± 0.4	848,469
Soil 6 mo. after application	Aflasafe	9	84.9 ± 0.0 *	14.1 ± 1.6 *	0.4 ± 0.1 ^ns^	794 ^ns^
	Control	18	65.6 ± 1.3	22.6 ± 2.1	0.8 ± 0.3	671

^a^ Number of fields. ^b^ Means of Aflasafe-treated variables with an asterisk (*) differ significantly from means of corresponding controls (Student’s *t*-test, α = 0.05). ^ns^ = not significant.

**Table 5:** Combined incidence of the four active ingredient atoxigenic African *Aspergillus flavus* vegetative compatibility groups (AAVs) of Aflasafe in soil before Aflasafe application, chili pepper at harvest, and soil collected six months after Aflasafe application during field efficacy trials in three states of Nigeria.

State	Mean Incidence of Aflasafe AAVs (%) ^a^					
	Soil before Application		Chili Pepper at Harvest		Soil 6 Months after Application	
	Control	Treated	Control	Treated	Control	Treated
Kano	10.4 ± 2.6	7.1 ± 2.5 ^ns^	6.9 ± 3.7	25.0 ± 8.1 *	7.2 ± 2.4	67.0 ± 5.4 *
Nasarawa	8.3 ± 1.8	8.8 ± 2.5 ^ns^	7.6 ± 4.3	17.5 ± 6.1 *	4.5 ± 2.7	64.6 ± 2.6 *
Oyo	10.4 ± 2.8	3.1 ± 1.7 *	9.4 ± 4.0	8.9 ± 1.3 ^ns^	4.9 ± 1.9	55.4 ± 4.2 *

^a^ An asterisk (*) indicates significant differences between treated and control samples (Student’s *t*-test, α = 0.05). ^ns^ = not significant.
